# Decoding innate lymphoid cells and innate-like lymphocytes in asthma: pathways to mechanisms and therapies

**DOI:** 10.1186/s12929-025-01142-w

**Published:** 2025-05-12

**Authors:** Christina Li-Ping Thio, Jheng-Syuan Shao, Chia-Hui Luo, Ya-Jen Chang

**Affiliations:** 1 Institute of Biomedical Sciences, Academia Sinica, No. 128 Academia Road, Section 2, Nankang, Taipei City, 115 Taiwan; 2 Taiwan International Graduate Program in Molecular Medicine, National Yang Ming Chiao Tung University and Academia Sinica, Taipei City, 115 Taiwan; 3 Institute of Translational Medicine and New Drug Development, China Medical University, Taichung City, 404 Taiwan

**Keywords:** Asthma, ILCs, Innate-like lymphocytes, Immune regulation, Therapeutic targets

## Abstract

Asthma is a chronic inflammatory lung disease driven by a complex interplay between innate and adaptive immune components. Among these, innate lymphoid cells (ILCs) and innate-like lymphocytes have emerged as crucial players in shaping the disease phenotype. Within the ILC family, group 2 ILCs (ILC2s), in particular, contribute significantly to type 2 inflammation through their rapid production of cytokines such as IL-5 and IL-13, promoting airway eosinophilia and airway hyperreactivity. On the other hand, innate-like lymphocytes such as invariant natural killer T (iNKT) cells can play either pathogenic or protective roles in asthma, depending on the stimuli and lung microenvironment. Regulatory mechanisms, including cytokine signaling, metabolic and dietary cues, and interactions with other immune cells, play critical roles in modulating their functions. In this review, we highlight current findings on the role of ILCs and innate-like lymphocytes in asthma development and pathogenesis. We also examine the underlying mechanisms regulating their function and their interplay with other immune cells. Finally, we explore current therapies targeting these cells and their effector cytokines for asthma management.

## Introduction

Asthma is a chronic respiratory disease affecting more than 300 million people of all ages worldwide [1]. The hallmark features of asthma include inflammation and airway constriction, causing symptoms like coughing, wheezing, chest tightness, and dyspnea. Inhaled corticosteroids (ICS) remain the mainstay therapy for asthma management, and can be administered individually or in combination with a controller medication such as long-acting beta agonists (LABA). Nevertheless, approximately 5–10% of patients are refractory to this conventional treatment, experiencing poor symptom control and frequent exacerbations [2]. These patients are categorized as having severe asthma and they account for a significant portion of asthma-related morbidity and healthcare expenditures [3].

In general, asthma can be classified into two endotypes: type 2 (T2)-high and T2-low/non-T2. T2-high asthma is defined by eosinophilic inflammation and elevated levels of T helper 2 (Th2) cytokines, mainly IL-4, IL-5, and IL-13 [4]. It can present as either an allergic or non-allergic phenotype and accounts for 50% of mild-to-moderate asthma, as well as a significant proportion of patients with severe asthma [5, 6]. T2-low asthma, on the other hand, is defined by the absence of T2-high features and can be further classified as either type 17 (T17)-high asthma, marked by neutrophilic inflammation and high circulating levels of IL-17 and IL-22, or T2-low and T17-low asthma [7]. This endotype is often severe and patients tend to be refractory to high doses of ICS [8].

Although initially thought to be mediated by the adaptive immune responses of B and T effector cells, recent studies have underscored the pivotal roles of innate lymphoid cells (ILCs) and innate-like lymphocytes in shaping the adaptive immunity and influencing disease outcomes in asthma. ILCs are non-B, non-T cells that lack antigen-specific receptors and exhibit phenotypes and functions similar to those of T cells. They are divided into five distinct subsets: natural killer (NK) cells, ILC1, ILC2, ILC3, and lymphoid tissue inducer cells (LTi) [9]. Among the ILCs, ILC2s are the most extensively studied and are particularly notable for their role in the pathogenesis of asthma. Innate-like lymphocytes include γδ T cells, invariant natural killer T (iNKT) cells, and mucosal-associated invariant T (MAIT) cells. These unconventional T cells are enriched in mucosal tissues such as the lungs and liver and respond rapidly to stress by secreting copious amounts of cytokines in a T cell antigen receptor (TCR)-dependent or independent manner [10]. In asthma, the role of innate-like lymphocytes remains controversial, with some studies reporting a protective function while others demonstrate a pathogenic effect.

In this review, we detail key characteristics of ILCs and innate-like lymphocytes, focusing primarily on ILC2s and iNKT cells, and examine their roles in the development of, or protection against, asthma in both human and mouse models. We explore the cellular and environmental triggers that influence the functions of these cells and highlight current existing therapies that could potentially be employed to target them.

## Characteristics of ILCs and innate-like lymphocytes

### ILCs

#### NK cells and ILC1s

NK cells and ILC1s are categorized as group 1 ILCs due to their shared ability to produce type 1 cytokines like interferon-gamma (IFN-γ) and tumor necrosis factor-alpha (TNF-α), as well as their dependence on the T-box transcription factor (T-bet) for lineage specification [11]. NK cells, identified as CD3^−^ CD56^+^ cells in humans and CD3^−^ NK1.1^+^ CD49b^+^ Nkp46^+^ cells in mice [12], make up 10–15% of lung lymphocytes [13]. Unlike ILC1s, the development of NK cells requires the transcription factor EOMES [14]. NK cells are highly cytotoxic and play a key role in eliminating cancer and virus-infected cells through the secretion of perforins and granzymes [15]. They recognize altered cell surface proteins via activating receptors like NKG2D and natural cytotoxicity receptors (NCRs), while inhibitory receptors such as NKG2A and killer immunoglobulin-like receptors (KIRs) modulate their activity [12, 16].

ILC1s, in contrast, are tissue-resident and respond to cytokines like IL-15, IL-12, and IL-18, particularly in response to intracellular pathogens [17]. Predominantly found in the intestinal intraepithelial compartment, ILC1s account for 25–50% of the total ILCs [18]. In humans, intraepithelial ILC1s have been described in gut and tonsil [19]. ILC1s express surface molecules that facilitate tissue retention, such as CD103, CD49a, and CD69 [20], and are crucial for defense against intracellular pathogens such as the parasite *Toxoplasma gondii.* They provide innate protection by producing IFN-γ to control the parasite burden [21]. ILC1s are also implicated in both cancer progression and protective antitumor responses, depending on the tumor type [22].

#### ILC2s

Identified in 2010 in the adipose tissue and small intestine of mice [23, 24], ILC2s are regarded as the innate counterparts of Th2 cells, requiring GATA3 for differentiation, maintenance, and function [25], and produce Th2-associated cytokines IL-13 and IL-5 [23]. In mice, ILC2s are present in the lungs, small intestine, adipose tissue, and skin, each exhibiting tissue-specific properties [26]. Lung ILC2s, or natural ILC2s, respond to IL-33 and express the IL-33 receptor ST2 [27]. In contrast, gut ILC2s become pro-inflammatory upon activation by IL-25 and are characterized by the expression of IL-25R and KLRG1 [28]. These gut ILC2s can migrate to the lungs during *Nippostrongylus brasiliensis* infection through sphingosine 1-phosphate (S1P) but will eventually revert to the natural ILC2 phenotype [28, 29]. While adipose tissue ILC2s are phenotypically more similar to lung ILC2s, dermal ILC2s express thymic stromal lymphopoietin (TSLP) receptors and are activated by IL-18 [26]. In humans, ILC2s are the predominant ILC population in the skin and can also be found in the blood, lungs, and tonsils, though they are rare in the small intestine [30, 31]. ILC2s have been shown to exhibit a memory phenotype upon exposure to allergens [32]. They also exhibit high functional plasticity, and can polarize to either ILC1- or ILC3-like cells upon stimulation with the respective activating cytokines [33–36]. ILC2s are associated with the development of many allergic diseases including atopic dermatitis, allergic rhinitis, and asthma [37, 38]. Nevertheless, they also have beneficial roles, such as promoting thermogenesis [39], aiding tissue repair through the production of amphiregulin [27], facilitating the expulsion of helminths [40], and enhancing antitumor immunity [41, 42].

#### ILC3s

ILC3s are the predominant ILC subset in the human lung and are also found in the intestine in both mice and humans [43–45]. They are a diverse population with at least four identified subsets: IL-17-producing Nkp46^−^ RORγt^+^ ILC3s [46], IL-22-producing Nkp46^+^ RORγt^+^ ILC3s [44], Nkp46^+^ RORγt^−^ ILC3s (‘ex-RORγt^+^’ ILC3s) [21], and LTi-like ILC3s [47]. The surface markers of ILC3s are highly heterogeneous, varying according to the specific subset and the tissue in which they reside [9]. In the lung, *Cd36* has been identified as a lung-specific marker for ILC3 [48]. ILC3s require the transcription factor retinoic acid receptor-related orphan receptor γt (RORγt) for development and produce IL-17A, IL-22, and granulocyte–macrophage colony-stimulating factor (GM-CSF) in response to IL-1β and IL-23 [49]. Notably, ILC3s exhibit phenotypic plasticity and can acquire an ILC1-like phenotype, a process regulated by the transcription factor c-Maf [50, 51]. Similar to ILC2s, lung ILC3s can acquire a memory-like phenotype following exposure to cigarette smoke and contribute to asthma exacerbation [52].

### Innate-like lymphocytes

#### iNKT cells

NKT cells are divided into type I and II based on differences in TCR usage, with iNKT cells belonging to the type I subset [53]. iNKT cells have a restricted TCRα chain (Vα14-Jα18 in mice and Vα24-Jα18 in humans), which mainly recognizes self-antigens and foreign lipid antigens presented by CD1d on antigen-presenting cells [54, 55]. They share common phenotypic and functional features with the conventional T cells and NK cells [56], and can express either CD4 or exhibit a double-negative (CD4^−^ CD8^−^) phenotype in both humans and mice [56]. There are three main iNKT cell subtypes: NKT1, NKT2, and NKT17, which primarily produce IFN-γ, IL-4, and IL-17A, respectively [57]. This cytokine profile enables iNKT cells to either suppress or amplify allergic responses. For example, iNKT cells activated by α-GalCer prior to ovalbumin (OVA) challenge have been shown to exert a suppressive effect, whereas iNKT cells activated by house dust mite (HDM) extracts during the OVA sensitization period enhance the adjuvant effects of HDM extracts, thereby amplifying inflammation [58, 59]. Their cytokine profile, along with their cognate interactions, orchestrates the functions of various immune cells, making them attractive targets for therapeutic intervention.

#### γδ T cells

γδ T cells are a specialized subset of T cells that express a γδ TCR instead of the conventional αβ TCR. They are classified by the TCR V gene usage, with various γ (γ2–5, γ8, γ9, and γ11) and δ (δ1–3) genes used for classification in humans, while distinct γ chains define subsets in mice [60, 61]. γδ T cells make up approximately 10% of total T cells in human peripheral blood mononuclear cells (PBMCs) but can be expanded at mucosal barriers and in peripheral non-lymphoid organs [62]. These cells recognize endogenous soluble and non-protein antigens, such as stress-induced molecules (MICA/B) and phospho-antigens, without requiring antigen processing and major histocompatibility complex (MHC) presentation [63]. Two major subsets have been defined in mice based on cytokine production: IL-17- and IFN-γ-producing subsets [64]. Their role in asthma is complex, as studies suggest they can have both protective and pathogenic effects in allergen-induced asthma [65, 66]. Specifically, γδ T cells promote allergic airway inflammation during a short-term challenge (4-day) with cockroach extract [66], but reverse airway hyperreactivity (AHR) after a long-term challenge (52-day) with OVA [65].

#### MAIT cells

MAIT cells express the MHC class I-related molecule MR1 and detect microbial metabolites derived from vitamin B_9_ or B_2_ (riboflavin) [67]. These metabolites, produced by bacteria and fungi capable of synthesizing riboflavin, enable MAIT cells to detect microbial infections [68]. MAIT cells feature a semi-invariant TCR, comprising a conserved Vα chain (Vα7.2 in humans and Vα19 in mice) paired with a limited range of Vβ chains (Vβ2/Vβ13 in humans and Vβ6/Vβ8 in mice) [69]. MAIT cells can respond to a variety of inflammatory stimuli, including IL-7, IL-12, IL-15, IL-18, and IL-23, through the expression of their corresponding cytokine receptors [62]. Upon activation, they rapidly produce cytokines such as IFN-γ, TNF-α, and IL-17 and cytotoxic substances such as perforin and granzyme, contributing to antimicrobial defense and inflammation [69]. MAIT cells have been implicated in several lung diseases, including asthma, chronic obstructive pulmonary disease (COPD), pneumonia, tuberculosis, and lung cancer [70, 71].

## ILCs and innate-like lymphocytes in asthma

Extensive evidence highlights the diverse roles of ILCs and innate-like lymphocytes in the pathogenesis of asthma across various inflammatory phenotypes [72, 73]. The importance of ILCs and innate-like lymphocytes in asthma development has been well-established in both asthmatic murine models [74, 75] and patients with asthma [72, 76].

### ILCs

Group 1 ILCs, including ILC1s and NK cells, exhibit complex roles in asthma. ILC1s are reduced in the blood of patients with allergic and non-allergic eosinophilic asthma [77], whereas their levels are elevated in the sputum of asthma patients with neutrophilic inflammation, where they are linked to inflammasome activation, IFN-γ, and TNF signaling [78]. ILC1s, together with ILC3s, induce M1 macrophage polarization through IFN-γ and IL-17A, and contribute to asthma pathogenesis [79]. NK cells, on the other hand, exhibit elevated frequencies and cytotoxic activity in both asthmatic adults and children, with distinct cytokine profiles observed, including increased IL-4 and reduced IFN-γ production [80–83]. In mice, NK cells exhibit dual roles: they promote eosinophilic inflammation and enhance Th2 response in allergic asthma models such as OVA and HDM [84, 85], but also provide protection against respiratory syncytial virus (RSV)-induced allergic airway inflammation through IFN-γ production [86]. In humans, NK cells are highly activated in severe asthma and help down-modulate the inflammatory response by inducing eosinophil apoptosis [87]. Collectively, these findings highlight the ambiguous roles of NK cells in asthma, highlighting the need for further investigation.

ILC2s are increased in the peripheral blood of asthma patients compared to healthy individuals or those with allergic rhinitis [88, 89]. Sputum analysis also revealed that allergen exposure increases ILC2 numbers in asthma patients [90]. Clinical studies highlighted a correlation between the alarmin cytokine IL-33 and ILC2s in peripheral blood [91] and bronchoalveolar lavage fluid (BALF) [92], influencing allergic asthma symptoms. Animal studies using asthma models (RSV, OVA, HDM, or fungi) demonstrated that epithelial alarmin-ILC2 interactions drive eosinophil infiltration, AHR, and mucus production [93–98]. Recent studies have revealed the diversity of human circulating ILC2s. Initially, human ILC2s were identified by their expression of CD127 (IL-7 receptor α-subunit), CD161, and CRTH2 (prostaglandin D2 receptor) [31]. However, subsequent research has identified some ILC2-like cells in human peripheral blood that lack CRTH2 expression but instead express KLRG1 [99–101]. Maho et al. further demonstrated that KLRG1^+^ ILCs can differentiate into other ILC subsets [100]. CRTH2^−^ ILC2s are enriched in the blood and BALF of patients with asthma [101], and another study discovered that the dynamic regulation of CRTH2 expression might be linked to the migration of human ILC2s into lung tissues. In mice, the accumulation of ILC2s and development of type 2 inflammation in the lung depend on CRTH2 [99]. Other ILC2 subsets, such as CCR10^+^ ILC2s, have been found to be enriched in the blood of both allergic and non-allergic severe asthmatic patients. CCR10^+^ ILC2s secrete minimal Th2 cytokines but possess ILC1-like features, including IFN-γ production [102]. In an allergen-induced asthma model, depletion of CCR10^+^ ILC2s resulted in the exacerbation of AHR [102]. These findings indicate the protective role of CCR10^+^ ILC2s in allergic inflammation.

Studies on the role of ILC3s in asthma remain limited. Notably, IL-17 has been reported to play a pertinent role in the pathogenesis of severe asthma and is associated with the recruitment and activation of neutrophils in the airways [103]. Human studies have also shown that patients with severe asthma exhibit higher numbers of IL-17-producing ILC3s in BALF compared to individuals with mild asthma or those without asthma [104]. Based on these observations, Kim et al. further demonstrated that high fat diet (HFD) up-regulated IL-1β expression, which potently stimulates and increases ILC3s in obese lungs. Furthermore, IL-17-producing ILC3s were found to be essential for the development of obesity-related AHR [104]. Collectively, these studies highlight the potential role of the ILC3-IL-17 axis in the pathogenesis of asthma.

### Innate-like lymphocytes

The role of iNKT cells in asthma has been extensively studied in various murine models, including those induced by allergens [105, 106], ozone [107, 108], and viruses [109]. Clinically, iNKT cells have been found in lung [110, 111], sputum [112], BALF [113], and sinus mucosa [114] samples from patients with asthma. However, significant variability exists in the reported numbers of iNKT cells in asthmatic patients. While some studies have observed an increase in iNKT cells, others have reported a decrease compared to healthy individuals. This variability likely reflects differences in patient populations, tissues analyzed, and detection methods. Several novel cytokines have been found to modulate iNKT cells, thereby contributing to the development of asthma. Notably, blood iNKT cells in asthmatic patients exhibit a Th2-like phenotype, with IL-4-producing iNKT cells potentially playing a critical role in modulating lung function [115]. Cytokines such as IL-25, IL-33 and TSLP activate iNKT cells, contributing to the development of AHR [116–118]. While murine models highlight the role of iNKT subsets in asthma, their relevance to human disease warrants further study.

Reports on the frequency of γδ T cells in asthma patients show varying results. Early studies demonstrated a decreased percentage of blood γδ T cells in asthmatic patients [119, 120]. In contrast, another study found no significant difference in γδ T cell levels between asthmatic patients and healthy controls across peripheral blood, induced sputum, or BALF [121]. γδ T cells are known to produce cytokines that contribute to the pathogenesis of neutrophilic asthma, such as IFN-γ and IL-17 [122, 123]. In allergic asthma patients, an increased frequency of IL-4-producing γδ T cells and a reduced frequency of IFN-γ-producing γδ T cells were observed compared to healthy controls [124]. Moreover, Krug et al. found an increased frequency of IL-5^+^ γδ T cells and IL-13^+^ γδ T cells in the BALF of asthmatic patients after segmental allergen challenge [125], suggesting a possible skew towards a Th2-type response and a potential involvement in allergic asthma. Overall, these studies underscore the complexity of the γδ T cell compartment in asthma and its potential significance in asthma pathogenesis.

Several studies have reported an association between MAIT cells and asthma. The frequency of MAIT cells is reduced in the peripheral blood, sputum, BALF, and lungs of asthmatic patients compared to healthy individuals [126–129]. In children with asthma, IL-17-producing MAIT cells have been observed to correlate with asthma severity [129]. Moreover, in severe asthma, the frequencies of MAIT cells, NK cells, ILC1s, ILC2s, and ILC3s show a positive correlation, indicating potential interactions between MAIT cells and these innate lymphoid cells [127]. In HDM and *Alternaria alternata*-induced mouse models, MAIT cells have been shown to regulate airway inflammation by suppressing ILC2 proliferation and activity [71]. Moreover, MAIT cells have also been found to protect against asthma by regulating the mucosal microbiota [130]. Collectively, the studies in both humans and mice reveal the involvement of MAIT cells in asthma pathogenesis.

## ILC and innate-like lymphocyte regulation and immune crosstalk in asthma

**Fig. 1:**
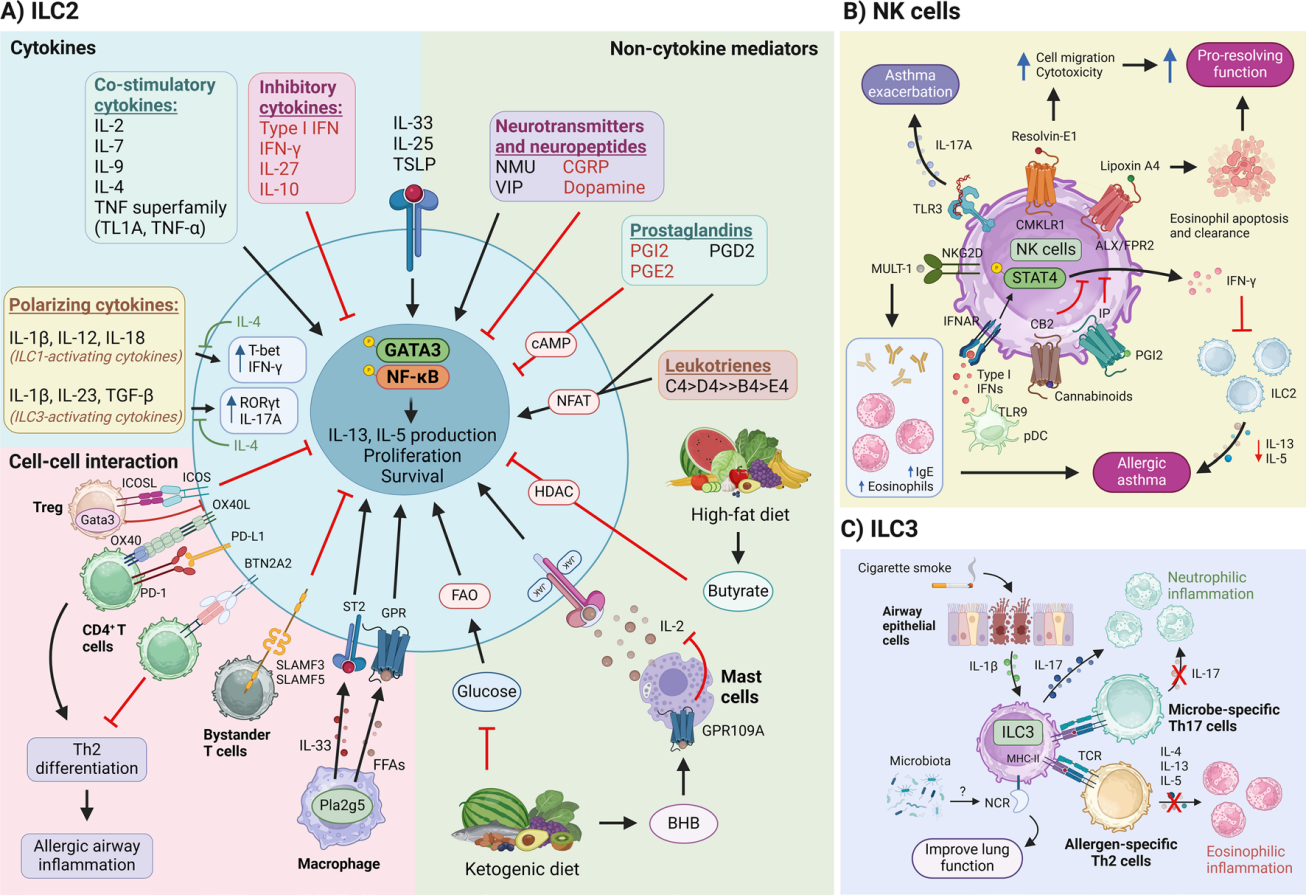
Modulation of ILC function in asthma. **A** ILC2 activation is influenced by cytokines and other mediators. IL-33, TSLP, and IL-25 are primary activators, while IL-2, IL-7, IL-9, IL-4, and TNF superfamily members act as co-factors. Meanwhile, type I/II IFNs, IL-27, and IL-10 inhibit ILC2s. Activation leads to GATA3 and NF-κB phosphorylation, driving IL-13 and IL-5 production, proliferation, and survival. IL-1β, IL-12, and IL-18 promote ILC1-like differentiation, marked by IFN-γ production and T-bet expression, while IL-1β, IL-23, and TGF-β drive polarization into IL-17A-producting ILC3-like cells. Notably, IL-4 counteracts these effects. Non-cytokine regulators, including neuropeptides (NMU, VIP, CGRP), prostaglandins (PGI2, PGE2, PGD2), and leukotrienes, can either inhibit or enhance ILC2 activation. Butyrate inhibits ILC2s via HDAC suppression, while BHB indirectly suppresses ILC2s by limiting IL-2 from mast cells. Tregs (ICOS-ICOSL) and SLAMF receptors inhibit ILC2s, while Pla2g5^+^ macrophages (FFAs, IL-33) activate ILC2s. ILC2s also influence CD4^+^ T cells via BTN2A2, PD-L1, and OX40L, promoting Th2 differentiation. Notably, GATA3-expressing Tregs inhibit OX40L expression on ILC2s. **B** Resolvin-E1 promotes NK cell migration and enhances cytotoxic abilities, while lipoxin A4 promotes eosinophil apoptosis and clearance, together boosting pro-resolving functions of NK cells. In contrast, cannabinoids and PGI2 inhibit IFN-γ production by NK cells, subsequently attenuating ILC2 function. Type I IFNs from pDCs drive NK cell IFN-γ production. NK cell pathogenicity involves TLR3-induced IL-17A production, worsening asthma. NKG2D^+^ NK cells, via MULT-1, elevate IgE and eosinophil levels, promoting allergic airway inflammation. **C** ILC3s drive neutrophilic inflammation via IL-17. Cigarette smoke induces IL-1β from airway epithelium, generating memory-like ILC3s that worsen neutrophilic asthma. Conversely, MHC-II engagement on ILC3s inhibits microbe-specific Th17 and allergen-specific Th2 cells, reducing neutrophilic and eosinophilic asthma, respectively. NCR^+^ ILC3s interact with the microbiome to enhance protective functions via an unknown mechanism

**Fig. 2:**
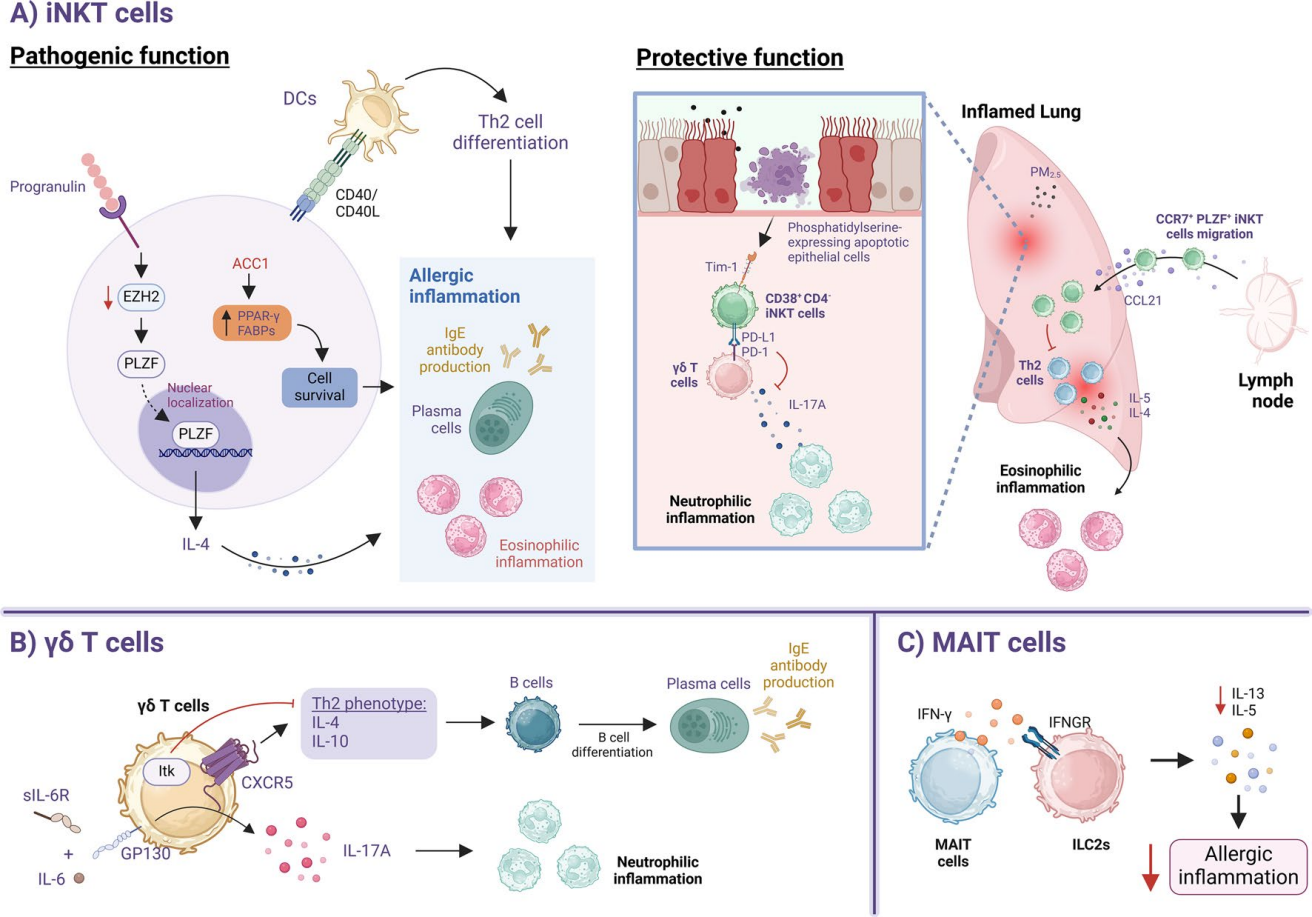
Regulation of the pathogenic or protective functions of innate-like T cells in asthma. **A** IL-4 secretion by iNKT cells promotes allergic inflammation, with progranulin enhancing IL-4 production by downregulating EZH2 expression, thereby facilitating PLZF translocation and subsequent IL-4 expression. CD40-CD40L interaction between iNKT cells and DCs promotes Th2 differentiation, contributing to allergic inflammation. ACC1 mediates de novo fatty acid synthesis by upregulating PPARγ and FABPs, which promotes iNKT cell survival and enhances their deleterious functions. The protective functions of iNKT cells have been demonstrated in a PM_2.5_ model, where phosphatidylserine-expressing apoptotic epithelial cells activate suppressive CD38^+^ CD4^−^ iNKT cells, leading to the upregulation of PD-L1 expression through interaction with Tim-1. PD-L1/PD-1 interaction between CD4^−^ iNKT cells and γδ T cells inhibits IL-17A production by γδ T cells, thereby reducing neutrophilic inflammation. CCL21 can recruit CCR7^+^ PLZF^+^ iNKT cells into inflamed lungs to restrain Th2 response and mitigate eosinophilic inflammation. **B** CXCR5^+^ γδ T cells in allergic inflammation exhibit a Th2 phenotype (producing IL-4 and IL-10), promoting antibody production. Notably, Itk suppresses their development. IL-17A-producing γδ T cells contribute to neutrophilic inflammation via IL-6 trans-signaling, where IL-6 forms a complex with soluble IL-6R (sIL-6R) and binds to the GP130 receptor, amplifying inflammation. **C** MAIT cells produce IFN-γ which suppresses ILC2 cytokine production, attenuating airway inflammation

Understanding the regulation of ILCs and innate-like lymphocytes is key to uncovering their role in driving asthma. Targeting these regulatory mechanisms offers potential for precise therapies to modulate inflammation and restore immune balance. This section examines how cytokines, non-cytokine mediators, and crosstalk with other effector cells influence the function of ILCs (Fig. 1) and innate-like lymphocytes (Fig. 2).

### ILCs

ILC2 proliferation and effector function can be regulated by various factors including cytokines, lipid mediators, neurotransmitters, dietary metabolites, and cellular crosstalk (Fig. 1A). Beyond the well-established role of epithelial-derived cytokines in activating ILC2s, additional co-stimulatory cytokines have been identified as key regulators of ILC2 activity. Both IL-7 and IL-2 work synergistically with IL-33 to increase ILC2 numbers and induce corticosteroid resistance in vitro [131], while IL-9 supports ILC2 proliferation and type 2 cytokine production [132]. Basophil-derived IL-4 promotes ILC2 effector function in papain-induced airway inflammation [133]. Furthermore, members of the TNF superfamily, TL1A and TNF-α, amplify ILC2-mediated airway inflammation by enhancing ILC2 survival, expansion, and cytokine production [134, 135]. Conversely, ILC2 activation and effector functions are directly counteracted by inhibitory cytokines such as type I and type II interferons. Our group has identified an indirect mechanism through which type I IFN suppresses lung ILC2 function by stimulating IFN-γ production by NK cells. Both type I and II IFNs inhibit ILC2 proliferation and type 2 cytokine production via the signal transducer and activator of transcription 1 (STAT1) signaling pathway [136]. Additionally, IL-27 directly inhibits ILC2 function through STAT1 [137], while IL-10, another anti-inflammatory cytokine, attenuates ILC2 activation and function in humans and mice [96, 138]. Interestingly, IL-10 specifically inhibits IL-33-mediated ILC2 activation but does not suppress ILC2 responses to IL-25 [96]. ILC2s also exhibit a high degree of plasticity, predominantly demonstrated in human systems, with cytokines driving their differentiation into ILC1- and ILC3-like cells, based on transcription factor expression and cytokine profiles [33, 139, 140]. Notably, this plasticity is inhibited by IL-4 [36]. Through this plasticity, ILC2s contribute to both allergic and non-allergic forms of asthma.

Non-cytokine factors also play a significant role in regulating ILC2 function. Earlier studies have highlighted the roles of leukotrienes, prostaglandins, and neuropeptides in modulating lung ILC2 activity. Elevated levels of leukotrienes have been detected in the sputum and plasma of individuals with asthma [141, 142], and preclinical studies have shown that leukotrienes synergize with IL-33 via the nuclear factor of activated T cells (NFAT) pathway to activate ILC2s during type 2 inflammation [143]. Conversely, prostaglandin I2 (PGI2) and prostaglandin E2 (PGE2) inhibit ILC2 function through the cyclic AMP (cAMP) pathway [144, 145], while prostaglandin D2 (PGD2) promotes ILC2 activity [146]. Neuroimmune regulation is equally crucial in modulating lung ILC2 function. Neuropeptides and neurotransmitters, such as vasoactive intestinal peptide (VIP) and neuromedin U, have been shown to activate ILC2s in mouse models of asthma [147, 148]. In contrast, calcitonin gene-related peptide (CGRP) and dopamine attenuate ILC2 effector functions [149, 150]. More recent studies have demonstrated the importance of calcium ion channels (Orai1) and iron in promoting ILC2 function by modulating ILC2 metabolism, leading to AHR and type 2 inflammation [151, 152]. Our group has particularly focused on metabolic factors, such as the short-chain fatty acid (SCFA) butyrate and the ketone body β-hydroxybutyrate (BHB). Butyrate, predominantly produced by the gut microbiome, directly inhibits ILC2 proliferation and type 2 cytokine production through inhibition of histone deacetylase (HDAC) signaling [153]. Although we did not identify the specific HDAC involved in this study, our recent findings in dermal ILC2s suggest that butyrate acts via HDAC3 [154]. A subsequent study further corroborated our findings, showing that butyrate inhibits ILC2-driven allergic airway inflammation by reducing IL-13 and IL-5 production while promoting IL-17A production by ILC2s [155]. Additionally, Karagiannis et al. reported that the ketogenic diet attenuates lung ILC2 function by impairing fatty acid metabolism [156]. BHB is one of the major ketone bodies produced during ketogenic diet feeding [157]. Building on this research, we demonstrated that BHB suppresses ILC2 function and mitigates allergic airway inflammation in the *Alternaria alternata* model [158]. Unlike butyrate, BHB does not act directly on ILC2 but instead targets mast cells, inhibiting IL-2 production via G protein-coupled receptor 109 A (GPR109A). Collectively, these studies underscore the potential impact of diet on asthma management.

ILC2s coordinate with various immune cells in a complex network of cellular crosstalk, influencing immune responses and contributing to asthma development. ILC2-derived IL-13 acts on dendritic cells (DCs) to potentiate Th2 effector cell generation [159]. ILC2s also regulate Th2 cell differentiation by expressing co-stimulatory molecules, such as OX40L and butyrophilin 2a2 (BTN2A2), as well as PD-L1, in response to epithelial-derived cytokines. BTN2A2 interaction with its receptor on T cells inhibits T cell effector function [160], while OX40L engagement with its receptor promotes Th2 cell expansion [161]. On the other hand, engagement of PD-L1 with PD-1 on T cells promotes GATA3 expression and IL-13 production by Th2 cells [162]. A recent study has shown that GATA3^hi^ regulatory T cells (Tregs) negatively regulate the availability of OX40L on ILC2s, thereby limiting the expansion of effector memory Th2 cells and attenuating airway inflammation [163]. In addition, interactions between ILC2s and bystander T cells in the mediastinal lymph node through signaling lymphocyte activation molecule 3 (SLAMF3) and SLAMF5 suppress ILC2-derived IL-13 production, which in turn inhibits DC activation and Th2 differentiation in the papain model of allergic asthma [164]. ILC2 function is further modulated through interactions with other lung immune cells. Group V phospholipase A_2_ (Pla2g5) regulates IL-33 and free fatty acid (FFA) production by lung macrophages, which, in turn, stimulates ILC2 activation and expansion upon exposure to *Alternaria alternata* [165]. Eosinophils play a reciprocal role in promoting ILC2 accumulation in the lungs through IL-4 and IL-13 [166]. Additionally, Tregs can inhibit ILC2 activity through inducible T cell co-stimulator (ICOS):ICOSL interaction [167].

Although the involvement of ILC1s in asthma has not been extensively studied, several reports have highlighted a dual role for NK cells in allergic asthma. NK cell protective function is differentially regulated by various eicosanoids, including resolvin-E1, prostaglandins, lipoxin-A4, and cannabinoid receptor 2 (CB2). Both resolvin-E1 and lipoxin-A4 enhance NK cell protective functions. Resolvin-E1 promotes NK cell migration and cytotoxicity, thereby attenuating allergic inflammation [168]. Similarly, lipoxin-A4 activates NK cells by interacting with pro-resolving ALX/FPR2 receptors, enhancing their ability to clear airway eosinophils via apoptosis [87]. In contrast, PGI2 and CB2 suppress NK cell activity. In PGI-deficient mice, an increase in IFN-γ-producing NK cells inhibits ILC2 function, reducing inflammation and highlighting an anti-inflammatory role for NK cells [169]. Likewise, the absence of the CB2 receptor increases IFN- γ-producing pulmonary NK cells, leading to reduced ILC2 numbers, airway eosinophilia, and lower type 2 cytokine production in response to HDM exposure [170]. Beyond eicosanoids, NK cell function can also be regulated by other factors, such as Toll-like receptors (TLRs). Notably, poly(I:C)-mediated TLR3 activation of NK cells stimulates IL-17A production, leading to asthma exacerbation [171]. Additionally, TLR9 can indirectly stimulate NK cell production of IFN-γ via plasmacytoid dendritic cell (pDC)-derived type I IFNs, which suppress ILC2s and alleviate airway inflammation [136]. Finally, NK cell activation through the activating receptor NKG2D, triggered by its ligand murine UL16 binding protein-like transcript (MULT-1) and granzyme B production, further promotes HDM-mediated allergic inflammatory responses [85] (Fig. 1B).

Research into the mechanisms regulating ILC3 function and their role in orchestrating immune responses in asthma remains limited. However, several recent studies have begun to address this gap. IL-1β is recognized as a key cytokine driving ILC3 activation and the subsequent production of IL-17A, which contributes to neutrophilic airway inflammation [172]. A recent study further reported that IL-1β production by airway epithelial cells is elevated in smokers and is associated with the development of memory-like ILC3s, which exacerbate asthma symptoms [52]. The positive association between NCR^+^ ILC3s and the gut microbiome has also been implicated, with a potential role for NCR^+^ ILC3s in improving lung function in severe asthmatics [173]. Additionally, ILC3s, through MHC-II interactions with allergen-specific Th2 cells and microbe-specific Th17 cells, limit eosinophilic and neutrophilic inflammation, respectively [174] (Fig. 1C).

### Innate-like lymphocytes

iNKT cells can play both protective and pathogenic roles in asthma (Fig. 2A). From a pathogenic perspective, iNKT cells are linked to the development of allergic asthma. A recent study reported that IL-4 production by lung iNKT cells is modulated by progranulin through the regulation of enhancer of zeste homolog 2 (EZH2) expression [175], a transcription activator previously shown to restrain pathogenic iNKT cell development [176]. In another study, de novo-fatty acid synthesis, mediated by acetyl-coA-carboxylase 1 (ACC1), promotes iNKT survival and exacerbates their deleterious effects in OVA and HDM models of allergic asthma [177]. iNKT cells can further enhance the immune response by upregulating the co-stimulatory molecule CD40L, which promotes DC maturation and subsequent CD4^+^ T cell differentiation [178]. Similarly, Deng et al. highlighted the potential importance of CD40-CD40L interactions between iNKT cells and DCs in promoting an asthma-like phenotype in the OVA model, using CD1d-deficient mice [179]. This highlights the critical role of iNKT cells in bridging innate and adaptive immunity in asthma pathogenesis.

While the aforementioned studies highlight the deleterious effects of iNKT cells, we and others have characterized a suppressive subset of iNKT cells and elucidated the regulatory pathways underlying their immunosuppressive functions. In an earlier study, Chuang et al. reported that influenza infection in neonatal mice preferentially expanded a suppressive CD4^−^ CD8^−^ iNKT population that highly expresses CD38 and can mitigate CD4^+^ T cell proliferation and the subsequent development of asthma [180]. Our study also identified a similar population that was induced by PM_2.5_ exposure in the absence of any glycolipid stimulation. Notably, we found that this subset is activated upon recognition of apoptotic cells through T-cell immunoglobulin and mucin domain 1 (TIM-1). This activation led to upregulation of PD-L1, which, through PD-1 signaling, suppressed the effector function of γδ T cells, resulting in reduced neutrophilic inflammation and attenuated AHR [181]. In a separate study, the chemokine CCL21 was shown to facilitate the migration of promyelocytic leukemia zinc finger protein (PLZF)^+^ iNKT cells to the lung, contributing to decreased airway resistance and increased asthma tolerance [182]. Nevertheless, further studies are required to delineate the exact mechanism by which these cells promote asthma tolerance.

In contrast to iNKT cells, the specific co-stimulatory mediators regulating γδ T cell and MAIT cell effector functions in the context of asthma remain poorly characterized. Nonetheless, studies have linked γδ T cells to the regulation of IgE production and neutrophilic asthma (Fig. 2B). Specifically, allergen-induced IL-6 trans-signaling promotes IL-17A production by γδ T cells, driving neutrophilic recruitment into the airways [66]. Consistently, asthmatic patients exhibiting elevated trans-signaling also present with increased neutrophilic and mixed granulocytic subtypes [66]. In non-allergic settings, PM_2.5_-driven neutrophilic inflammation is also mediated by γδ T cells in an IL-17A-dependent manner [181]. Crosstalk between γδ T cells and B cells has also been reported, where IL-4 and IL-10-producing CXCR5^+^ γδ T cells facilitate B cell antibody production [183]. Development of these Th2 cytokine-producing γδ T cells is negatively regulated by Itk, as Itk-deficient γδ T cells produce more Th2 cytokines, accompanied by increased IgE levels [184]. This effect is subset-specific: Vγ1 γδ T cells promote IgE production, whereas Vγ4 γδ T cells have an opposing function [185]. MAIT cells, on the other hand, can produce IFN-γ, which has been shown to suppress ILC2 function in *Alternaria alternata*-induced allergic airway inflammation [186] (Fig. 2C).

## Potential therapeutic approaches targeting ILCs and innate-like lymphocytes

With growing research elucidating the roles of ILCs and innate-like lymphocytes in asthma, these immune cell populations have emerged as promising therapeutic targets. Here, we will discuss recent findings in the therapeutic manipulation of ILCs and innate-like lymphocytes in both animal models and human studies (Summarized in Table 1).

**Table 1:** Potential therapies for asthma and their mechanisms of action

Drugs	Mechanism of action	Effects	Progression status	References
Corticosteroids				
Glucocorticoids	Inhibits MEK/JAK-STAT signaling pathways	Suppresses IL-5, IL-13 and IL-9 production from ILC2	FDA approved	[187]
Alarmins				
Itepekimab	Binds to IL-33	Inhibits IL-33/ST2 signaling	Phase 3 trial for COPD Phase 2 trial for asthma	[188] [189]
Astegolimab	Binds to IL-33 receptor, ST2	Inhibits IL-33/ST2 signaling	Phase 2 trial for asthma	[190]
Tozorakimab	Binds to IL-33	Inhibits IL-33/ST2 signaling	Phase 3 trial for COPD and viral lung infection	[192, 193]
Tezepelumab	Binds to TSLP	Inhibits TSLP	FDA approved	[194, 195]
Effector cytokines				
Dupilumab	Binds to IL-4Ra	Inhibits IL-4R signaling induced by IL-4 and IL-13	FDA approved	[197]
Mepolizumab	Binds to IL-5	Suppresses IL-5R signaling	FDA approved	[201]
Reslizumab	Binds to IL-5	Suppresses IL-5R signaling	FDA approved	[202]
Benralizumab	Binds to IL-5Ra	Inhibits IL-5R signaling	FDA approved	[203]
Bronchodilators				
β2-agonists	Inhibits ILC2 proliferation cell-intrinsically	Negatively regulates ILC2 proliferation and effector function	FDA approved	[208]
Muscarinic antagonists	Attenuates ILC2 function by suppressing IL- 4 production from basophils	Suppresses ILC2 proliferation and cytokine production	FDA approved	[209, 210]
Montelukast	Blocks cysteinyl leukotriene 1 receptor	Suppresses ILC2 cytokine production	FDA approved	[211, 212]
Zafirlukast	Blocks cysteinyl leukotriene 1 receptor	Inhibits cysteinyl leukotriene 1 receptor signaling	FDA approved	[213]
Signaling pathways				
PF06651600	Blocks JAK3 kinase	Suppresses ILC2 survival, proliferation, and cytokine production	FDA approved	[214]
2-methoxyestradiol	Inhibits HIF-1α at the translational level	Suppresses ILC2 proliferation and cytokine production	Phase 2 trial for ovarian and prostate cancer	[215]
Glycolipids				
NU–α-GalCer	Stronger interaction with CD1d	Increases protective CD38^hi^ iNKT cells	Preclinical development	[180]
α-LacCer	Competes with α-GalCer for CD1d binding	Suppresses α-GalCer-induced IL-4 and IFN-γ production by iNKT cells	Preclinical development	[219]
sp2-iminoglycolipids	Competes with α-GalCer for CD1d binding	Suppresses α-GalCer-induced IL-2 secretion by iNKT cells	Preclinical development	[220, 221]
Other potential approaches				
Vitamin D3	Promotes Blimp-1 expression in ILC2s	Promotes IL-10 production from ILC2s	FDA approved	[222]
Yoda1	Activates Piezo1 channel	Reduces ILC2 oxidative metabolism and inhibits its cytokine production and proliferation	Preclinical development	[223]
Anti-CD226	Binds to CD226	Reduces ILC2 cytokine secretion and proliferation	Preclinical development	[224]
CD47Fc	Activates SIRPα	Reduces ILC2 cytokine production and proliferation	Preclinical development	[225]

### Corticosteroids

Glucocorticoids, widely used anti-inflammatory agents in asthma, have been shown to reduce ILC2 levels in asthmatic patients with allergic rhinitis. Additionally, glucocorticoids inhibit ILC2 function by suppressing IL-5, IL-13, and IL-9 production via MEK/JAK-STAT signaling pathways, as demonstrated in studies using ILC2s sorted from asthma patients’ PBMCs [187].

### Alarmins

ILC2 activity, survival, and cytokine production are regulated by alarmins such as IL-33, TSLP, and IL-25. There are several drugs that target these ILC2 activators. Itepekimab, a monoclonal antibody targeting IL-33, demonstrated improved lung function in patients with COPD during a phase 3 trial [188]. Additionally, a phase 2 trial revealed its efficacy in improving lung function in individuals with moderate-to-severe asthma [189]. Astegolimab is an anti-ST2, the IL-33 receptor, monoclonal antibody. A phase 2 trial for astegolimab reported a reduced asthma exacerbation rate (AER) in asthmatic patients, and is currently undergoing phase 3 trials for COPD [190]. However, in a phase 2 trial, astegolimab did not show significant improvement in disease outcomes for patients with atopic dermatitis [191]. Tozorakimab is an anti-IL-33 monoclonal antibody currently undergoing a phase 3 clinical trial to assess its efficacy in COPD and in patients hospitalized with viral lower respiratory tract infections [192, 193]. Tezepelumab is a monoclonal antibody that blocks TSLP. A phase 3 trial showed that patients with severe asthma experienced fewer exacerbations and improved lung function. Tezepelumab has already been approved for the treatment of asthma [194, 195]. On the other hand, drugs targeting IL-25 have not yet progressed to clinical trials. Although risankizumab, a monoclonal antibody targeting the ILC3 activator IL-23, was approved for the treatment in psoriasis, it failed to show significant improvement in severe asthma in a phase 2 trial [196].

### Effector cytokines

Dupilumab, a monoclonal antibody that targets the IL-4 receptor, has been approved for the treatment of eosinophilic esophagitis. A phase 3 trial demonstrated significant improvements in lung function and asthma control in patients with severe asthma, leading to FDA approval for its use in moderate and severe asthma patients [197]. The drug is also undergoing clinical trials for COPD [198, 199]. Mepolizumab and reslizumab, monoclonal antibodies targeting IL-5, are approved for asthma treatment, with their use in COPD currently in a phase 3 trial [200–202]. Benralizumab, a monoclonal antibody against the IL-5 receptor, showed improvements in clinical outcomes for asthma and COPD patients in a phase 2 trial [203]. Although drugs targeting IL-13 are under development, results have been disappointing so far [204]. Ixekizumab and secukinumab, which target the ILC3-related cytokine IL-17, are approved for psoriasis treatment. However, secukinumab failed to improve asthma control, as indicated by the Asthma Control Questionnaire (ACQ) scores in patients with uncontrolled asthma, leading to its termination (ClinicalTrials.gov Identifier: NCT01478360) [205, 206]. Brodalumab, a monoclonal antibody targeting the interleukin-17 receptor, has also been approved for psoriasis but did not improve asthma outcomes in a phase 2 trial [207].

### Bronchodilators

In addition to their established role as bronchodilators in asthma treatment, β2-agonists, muscarinic antagonists, and leukotriene receptor antagonists have been shown to regulate ILC2 function. β2-agonists, commonly used to relax airway smooth muscles, have been found to negatively regulate ILC2 proliferation and effector function [208]. Muscarinic antagonists, traditionally used to alleviate bronchoconstriction, also demonstrate inhibitory effects on ILC2 activity [209, 210]. Montelukast and zafirlukast are both cysteinyl leukotriene 1 (CysLT1) receptor antagonists approved for asthma management. Notably, montelukast has been shown to suppress ILC2 activity [211–213].

### Signaling pathways

JAK3 inhibitors have been shown to suppress ILC2 survival, proliferation, and cytokine production, thereby alleviating ILC2-driven asthma in murine models [214]. Moreover, ILC2 activation and function are highly dependent on cellular metabolic processes. Inhibiting hypoxia-inducible factor 1-alpha (HIF-1α) or disrupting glycolytic metabolism has been demonstrated to suppress ILC2 activity and mitigate airway inflammation in mouse models of asthma [215].

### Glycolipids

iNKT cells play a complex role in asthma, with certain subsets contributing to AHR and allergic inflammation, while others provide a protective effect. Activation of pulmonary iNKT cells by glycolipid antigens, such as α-GalCer and asperamide B, has been shown to induce AHR and eosinophilic inflammation in mice [216, 217]. However, other studies have shown that α-GalCer activation of iNKT cells can suppress AHR and allergic inflammation in an OVA-induced mouse model of asthma [218]. CD38^hi^ iNKT cells, a specific subset of iNKT cells, have been shown to ameliorate the development of AHR in mice. Moreover, treatment with NU–α-GalCer can increase the number of CD38^hi^ iNKT cells in the lungs and protect against allergen-induced AHR [180]. Additionally, α-LacCer, a weaker activator of iNKT cells compared to α-GalCer, can reduce AHR induced by both α-GalCer and glycosphingolipid (GSL)-1 [219]. Efforts to enhance therapeutic outcomes by developing alternative glycolipids have also been made, including the recent development of sp^2^-iminoglycolipids with enhanced biostability [220, 221]. These findings suggest that controlled activation of iNKT cells, potentially by using different glycolipid antigens and promoting regulatory subsets, may help reduce allergic airway inflammation.

### Other potential approaches

Vitamin D3 supplementation has been associated with less severe asthma in both humans and murine models, with its effects attributed to the modulation of ILC2 function [222]. Piezo1, a calcium ion channel, plays a crucial role in regulating ILC2 activity and AHR in asthma. In murine models, activation of Piezo1 using Yoda1 treatment significantly reduced ILC2 cytokine secretion, proliferation, and survival, leading to diminished AHR and lung inflammation [223]. Additionally, targeting CD226 has also shown potential in asthma therapy. In a mouse model, administration of an anti-CD226 antibody effectively reduced cytokine production by ILC2s and alleviated AHR triggered by IL-33 or *Alternaria alternata* [224]. Signal regulatory protein alpha (SIRPα), an inhibitory receptor that regulates ILC2 effector functions and AHR, has been shown to suppress ILC2 proliferation and cytokine secretion when engaged with CD47Fc, thereby mitigating AHR and lung inflammation in murine models [225].

## Conclusion

In conclusion, ILCs and innate-like lymphocytes are key regulators of immune responses in asthma, responding rapidly to environmental cues and allergen exposure. Their ability to amplify or dampen inflammation, depending on the microenvironment, highlights their functional plasticity and potential as therapeutic targets. However, the dynamics of their frequency, cytokine milieu, and receptor expression in the asthmatic lung remain unclear, including whether they shift from protective to pathogenic roles during disease progression. Future studies should focus on characterizing ILC subsets, their interplay with other immune cells, and developing tools to manipulate these cells in asthma models. Advancing our understanding of their biology could pave the way for novel immunotherapies to better manage or prevent asthma.

## Data Availability

Not applicable.

## Abbreviations

ACC1: Acetyl-coA-carboxylase 1
ACQ: Asthma Control Questionnaire
AHR: Airway hyperreactivity
BALF: Bronchoalveolar lavage fluid
BCL-3: B-cell lymphoma 3
BHB: β-Hydroxybutyrate
BTN2A2: Butyrophilin 2a2
cAMP: Cyclic AMP
CB2: Cannabinoid receptor 2
CGRP: Calcitonin gene-related peptide
CMKLR1: Chemerin chemokine-like receptor 1
COPD: Chronic obstructive pulmonary disease
CysLT1: Cysteinyl leukotriene 1
DCs: Dendritic cells
EZH2: Enhancer of zeste homolog 2
FABPs: Fatty acid binding proteins
FAO: Fatty acid oxidation
FFA: Free fatty acid
FPR2: Formyl peptide receptor 2
GATA3: GATA binding protein 3
GM-CSF: Granulocyte–macrophage colony-stimulating factor
GPR109A: G protein-coupled receptor 109 A
GSL-1: Glycosphingolipid
HDAC: Histone deacetylase
HDM: House dust mite
HFD: High fat diet
HIF-1α: Hypoxia-inducible factor 1-alpha
ICOS: Inducible T cell co-stimulator
ICS: Inhaled corticosteroid
IFN: Interferon
IFNAR: Interferon-α/β receptor
IFN-g: Interferon-gamma
ILCs: Innate lymphoid cells
ILC1: Group 1 innate lymphoid cells
ILC2: Group 2 innate lymphoid cells
ILC3: Group 3 innate lymphoid cells
iNKT: Invariant natural killer T cells
KIRs: Killer immunoglobulin-like receptors
LABA: Long-acting beta agonists
LTi: Lymphoid tissue inducer cells
MAIT: Mucosal-associated invariant T cells
MHC-II: Major histocompatibility complex class II
MULT-1: Murine UL16 binding protein-like transcript
NCRs: Natural cytotoxicity receptors
NFAT: Nuclear factor of activated T cells
NF-κB: Nuclear factor kappa B
NK cells: Natural killer cells
NKG2D: Natural killer group 2, member D
NMU: Neuromedin U
OVA: Ovalbumin
PBMC: Peripheral blood mononuclear cells
PD-1: Programmed cell death protein 1
pDC: Plasmacytoid DC
PD-L1: Programmed death-ligand 1
PGD2: Prostaglandin D2
PGE2: Prostaglandin E2
PGI2: Prostaglandin I2
Pla2g5: Group V phospholipase A_2_
PLZF: Promyelocytic leukemia zinc finger protein
PPAR-γ: Peroxisome proliferator-activated receptor gamma
RORgt: Retinoic acid receptor-related orphan receptor γt
RSV: Respiratory syncytial virus
SCFA: Short-chain fatty acid
SIRPα: Signal regulatory protein alpha
SLAMF: Signaling lymphocyte activation molecule family
STAT1: Signal transducer and activator of transcription 1
T2: Type 2
T17: Type 17
T-bet: T-box transcription factor
TCR: T cell antigen receptor
Th2: T helper 2
TIM1: T-cell immunoglobulin and mucin domain 1
TLRs: Toll-like receptors
TNF-a: Tumor necrosis factor-alpha
Treg: Regulatory T cells
TSLP: Thymic stromal lymphopoietin
VIP: Vasoactive intestinal peptide
